# Nanosized tetragonal β-FeSe phase obtained by mechanical alloying: structural, microstructural, magnetic and electrical characterization†

**DOI:** 10.1039/c7ra13473h

**Published:** 2018-02-20

**Authors:** K. F. Ulbrich, C. E. M. Campos

**Affiliations:** Departamento de Física, Universidade Federal de Santa Catarina Campus Trindade 88 040-900 Florianópolis Santa Catarina – SC Brazil carlos.campos@ufsc.br

## Abstract

Nanocrystalline tetragonal β-FeSe phase was prepared mechanochemically using ball milling procedures in an inert atmosphere, starting from Fe_*x*_Se powder mixtures with *x* = 1.00, 1.25 and 1.50, with *x* = 1.25 and 1.50 leading to more than 93% of pure phase after annealing at 400 °C for 1 hour under vacuum. X-ray powder diffraction provides information on phase formation and phase transitions with milling time and temperature. The Rietveld method was used to refine the crystal structure, including the *z* coordinate of Se and occupancies, to determine the microstructure and to assess the amount of contaminant phases observed. Lattice contraction is found in the *ab*-plane more than along the *c*-axis, the small average size of crystalline domains (<22 nm) and the high microstrain (>1%) indicate the formation of highly strained nanoparticles. Magnetic and electrical characterization showed a poor superconductivity at 4 K and semiconducting properties only for thermally treated samples. These observations are explained by the presence of ferromagnetic impurity phases (residual Fe, hexagonal δ-FeSe phase and monoclinic Fe_3_Se_4_), but other effects caused by the mechanochemical synthesis must be considered, such as small average size, large/non-uniform size distribution and high microstrain of the nanosized tetragonal β-FeSe phase. The increase of the β-FeSe phase content with increasing storage time (ageing) above a few days to months in air, at RT and in the dark was observed for all as-milled samples. Preliminary data on the ageing effect are shown while a systematic study on this is in progress and will be presented elsewhere.

## Abstract 2

We describe obtaining about 30% of tetragonal FeSe phase prepared by mechanically-alloying Fe and Se powders for 3 h, size–strain analysis as well as electrical and magnetic characterization.

## Graphical abstract

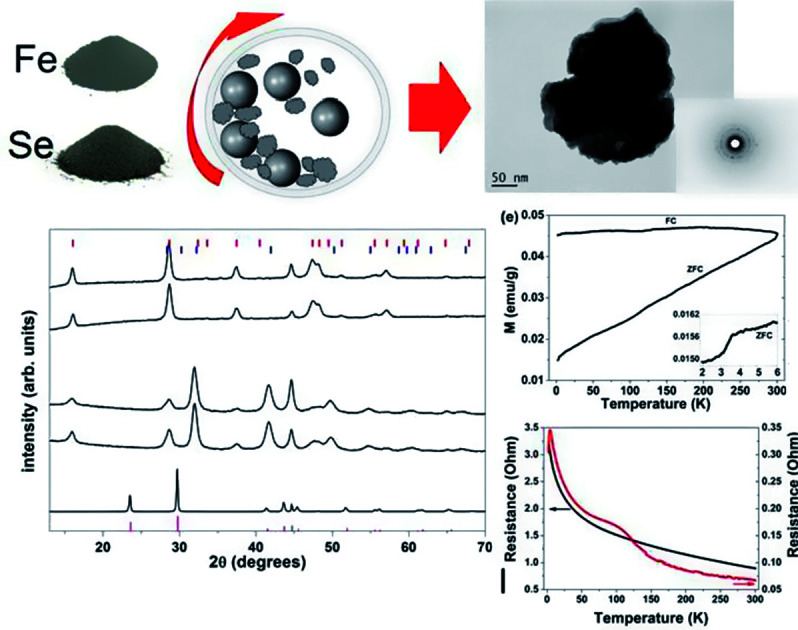

## Introduction

Iron selenides are found in several crystalline forms: β-FeSe phase with tetragonal PbO-like structure, δ-FeSe phase with hexagonal NiAs-type structure, a FeSe_2_ phase that has an orthorhombic marcasite structure, the hexagonal Fe_7_Se_8_ phase, which is ferrimagnetic with a Curie temperature of 125 K, and the monoclinic Fe_3_Se_4_ phase that is ferrimagnetic with *T*_Neel_ = 320 K.^1^ Since the recent discovery of superconductivity of tetragonal β-FeSe with critical temperature *T*_c_ of ∼8 K,^2^ a large number of studies have been conducted showing that superconductivity is linked to significant selenium deficiency.^3–7^*T*_c_ values can greatly increase up to 37 K and 55 K under high pressure,^8–11^ and 65 K was reported for monolayer films on SrTiO_3_ substrates.^12^ These high *T*_c_'s allied with the advantages such as low cost, and low toxicity of starting materials compared to FeAs-based superconductors^13,14^ suggest that FeSe has a great potential for practical applications. Some recent review articles have presented results mainly focused on superconductive properties, but photocatalytic activity, electrochemical sensing, and fuel cell activity of FeSe have been also observed.^15–23^ These reviews have showed that FeSe can be produced by solid state reactions, solution chemistry routes, chemical vapor deposition, spray pyrolysis and chemical vapor transport, but many efforts have been done to obtain FeSe using mechanical alloying assisted by sintering process.^15^ Many sintering parameters can influence the phase transition process of Fe–Se system, including the sintering temperature,^16^ pressure,^17^ and chemical compositions.^18^ Slight changing in sintering parameters and chemical composition can induce the appearing of non-superconducting hexagonal δ-FeSe (and/or Fe_7_Se_8_) phases, which decrease the superconducting volume weakening the intergrain connections, reducing the *T*_c_ value of FeSe samples by varying the chemical composition of β-FeSe phase.^17^ In fact, it is very hard to avoid the appearance of secondary phase during the preparation of FeSe bulks and wires,^19–22^ even single crystals,^23^ but these impurities do not avoid the superconducting applications.

Besides some authors claimed to have optimized the production of the β-FeSe by mechanical alloying, always sintering process is need to obtain it in a significant amount.^24–26^ When planetary ball mill was used with milling times as longer as 80 hours no reaction between Fe and Se was observed^27^ and when high-energy ball milling was applied no significant signal of the β-FeSe phase was reported, only after high temperature sintering process through long times it was observed, generally coexisting with other non-superconducting phases.^28^

In this paper, we report on the obtaining of the tetragonal β-FeSe phase by mechanochemical methods. Three compositions (Fe_1.00_Se, Fe_1.25_Se and Fe_1.50_Se) were tested leading up to 29% of the tetragonal β-FeSe phase, with only 3 h of mechanical alloying using high-energy ball mill, increasing up to 93% after thermal treatments at 400 °C for 1 hour under vacuum. X-ray Powder Diffraction (XRPD), Differential Scanning Calorimetry (DSC), Transmission Electron Microscopy (TEM) and Physical Property Measurement System (PPMS) were used to characterize the structural, thermal, microstructural, electrical and magnetic properties of the as-milled and thermal treated samples.

## Experimental section

### Solid state synthesis

Elemental Fe (Sigma-Aldrich 97% purity −325 mesh) and Se (Sigma-Aldrich 99.5% purity −100 mesh) powders were used with nominal compositions Fe_*x*_Se (*x* = 1.00, 1.25 and 1.50), which were sealed together with nine stain steel balls (three with an average diameter of 12.7 mm and six with average diameter of 6.35 mm) in a 65 ml cylindrical stainless steel container under argon atmosphere. The ball-to-powder weight ratio was 10 : 1. The container was mounted on a SPEX Mixer/mill, model 8000D, and milling was performed at room temperature. The first synthesis of the Fe_1.00_Se sample was interrupted after 3, 6, 9, 12, 15, 18, 21 and 24 hours of milling and, at each step, a small portion of the sample was collected for *ex situ* characterization (see ESI Fig. SI-1†). For the Fe_1.25_Se and Fe_1.50_Se samples the synthesis was done only for 3 hours. Second batches of all compositions were produced to confirm the reproducibility of the synthesis. The second batch of the Fe_1.0_Se sample was produced using the same milling jar (soft cleaned) of the first batch and small portion of the sample was collected after each 1 h up to 5 h, in order to test shorter milling times, resulting mainly in the hexagonal δ-FeSe and some residual iron. This effect can be explained by the effect of stopping milling before β-FeSe formation and/or because the soft cleaning of the container let some seeds of the δ-FeSe phase on the jar walls favoring its formation. A third batch of the Fe_1.0_Se sample was produced under the same conditions, using a cleaned container, and 29% of β-FeSe phase was found. Although coating of the jar and the milling tools with the desired material can be recommended to reduce the contamination effects by the milling tools,^29^ in this case it caused an undesired result.

The samples milled for 3 hours were thermal treated up to 400 °C (Fe_1.25_Se and Fe_1.50_Se) and 500 °C (Fe_1.00_Se) with heating rates of 10 °C min^−1^ and isotherm at maximum temperature for 1 hour using the Anton Paar Camera model HTK-16 equipped with a platinum filament as the sample holder. Thermal treatments were performed in vacuum better than 10^−5^ torr. All samples named thermal treated in this work are samples recovered from the HTK-16 camera.

### Characterization

X-ray powder diffraction (XRPD), Differential Scanning Calorimetry (DSC), transmission electron microscopy (TEM), and variable-temperature electrical and magnetic measurements were used to characterize the as-milled and thermally treated samples, including the full stoichiometric, structural and microstructural aspects, such as size and strain analysis.

### X-Ray powder diffraction

XRPD experiments were performed on powders of the Fe_*x*_Se materials (as-milled and thermal treated), deposited in the hollow of silicon zero background sample holder, 0.2 mm deep, as well as in deeper stainless steel holders. Diffraction data were collected on a vertical-scan Panalytical Xpert Multi-Purpose diffractometer operating in *θ*:*θ* mode, equipped with a linear position-sensitive Xcelerator detector, primary and secondary beam Soller slits of 0.04 rad, 1° divergent slits and Ni-filtered Cu-Kα radiation (*λ* = 1.5418 Å). Generator setting: 45 kV, 40 mA. Variable temperature XRPD patterns were collected using a HTK-16 camera according to the conditions described in the previous section. The structural refinement was carried out by the Rietveld method using TOPAS^30^ as follows: the background was modeled by a (11^th^ order) polynomial function of the Chebyshev type, peak profiles were described by the fundamental parameters approach,^31^ an isotropic Lorentzian broadening of 1/cos *θ* dependence to account to size effects and an isotropic (tan *θ* dependent) Gaussian broadening term to account for microstrain effects. Refinable isotropic thermal factors were individually attributed to all atoms and occupation of Fe and Se and *z* coordinates of Se atoms were also refined, reducing the agreement factors up to 28%.

### Calorimetry experiments

Differential Scanning Calorimetry (DSC) was employed to measure two successive heating and cooling runs for the as-milled Fe_1.00_Se sample (in hermetic aluminum pans) from 0 °C to 450 °C, with a heating rate of 10 °C min^−1^ and N_2_ gas flow/flux in a DSC cell, model Q2000, manufactured by TA instruments.

### Transmission electron microscopy

TEM imaging and Selected Area Electron Diffraction (SAED) measurements were carried out in a JEOL JEM-1011 transmission electron microscope with an accelerating voltage of 100 kV.

### Magnetic and electrical measurements

The magnetization measurements in zero-field-cooling (ZFC) and field-cooling (FC) curves conditions at an applied field of 10 Oe, as well as hysteresis curves up to magnetic fields of 90 kOe (9 T) with different temperatures (2 K and 300 K) were carried out with a Physical Properties Measurement System PPMS (Quantum Design, Dynacool). The four-probe resistance measurements were performed in the PPMS. The as-milled samples were cold pressed in regular pellets and some of them were annealed at 400 °C for one hour.

## Results and discussion

### Samples chemistry

**Fig. 1:**
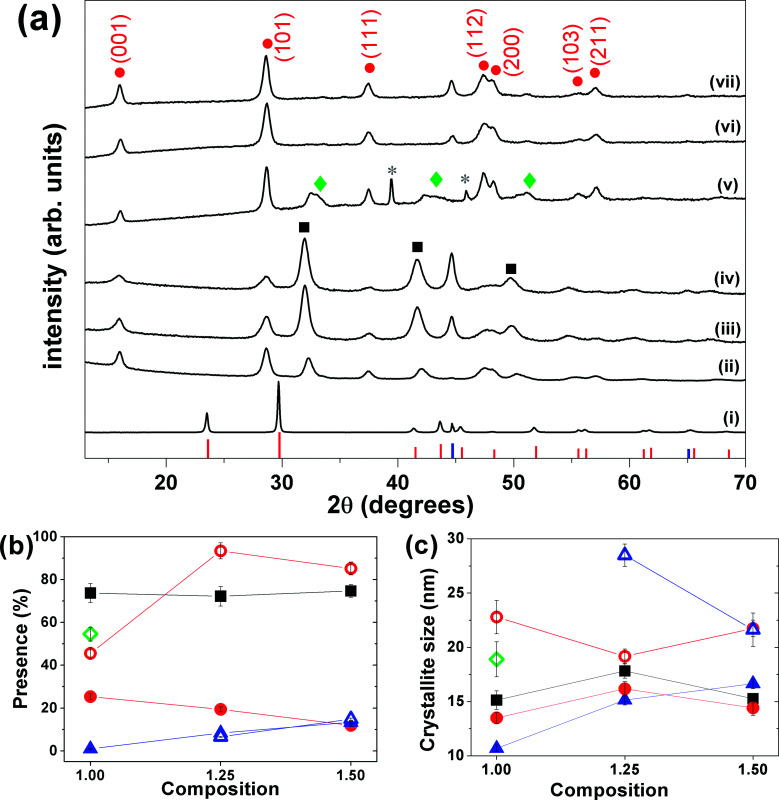
(a) XRPD patterns of (i) physical mixture Fe/Se (no milling), (ii) Fe_1.00_Se, (iii) Fe_1.25_Se and (iv) Fe_1.50_Se samples milled for 3 hours. XRPD patterns (v), (vi) and (vii) of the thermal treated are organized in the same sequence of the as-milled samples. The red, black and green symbols represent the peak positions of β-FeSe (ICSD 169251), δ-FeSe (ICSD 53542) and Fe_3_Se_4_ (ICSD 633477) phases, respectively. The pink and blue symbols represent the peak positions and relative intensity of trigonal Se (ICSD 53801) and cubic Fe (ICSD 53802). (b) Phase fractions and (c) average crystallite size for δ-FeSe, β-FeSe and residual Fe phases *versus* composition. In the last two panels the red, black and green symbols represent the values for β-FeSe, δ-FeSe and Fe_3_Se_4_ phases, respectively, while the solid symbols represent the values for the as-milled samples and the open ones represent those for the thermal treated samples.

Tetragonal β-FeSe NPs were easily formed by mechanical alloying iron and selenium powders in different molar ratios. X-ray diffraction patterns collected on the reaction products, before and after milling, show that with only 3 hours of milling the formation of the β-FeSe phase occurs accompanied by non-superconducting phases (hexagonal δ-FeSe for most compositions and batches tested), which remains stable and grows on the post-annealed sample (*T* = 400 or 500 °C for 1 h under vacuum) (see Fig. 1). For longer milling times the β-FeSe phase was no more observed and the hexagonal δ-FeSe remains as the unique phase up to 24 h (for *x* = 1.00) (see Fig. SI-1 in the ESI† file for details).

Despite the pattern similarity at 3 h milling for different *x* values (Fig. 1), a careful analysis indicated that the as-milled *x* = 1.00 sample provided the β-FeSe richest material, sanctioning the quantitative, and selective, transformation of the reactants into the desired alloy. For *x* = 1.25 and 1.50 materials, larger amounts of residual bcc-Fe are present, being iron in slight excess to the reaction stoichiometry; differently, samples prepared by equimolar amounts of Fe and Se (*x* = 1.00), show Bragg peaks of the Fe_3_Se_4_ phase, which progressively forming upon increasing of storage time (ageing) above few days in air, at RT and in the dark (see Fig. 1 and SI-2(b) and (c)†).

The Rietveld-based quantitative analysis (Fig. 1(b)) of the *x* = 1.00 mixture (3 h) provided the β-FeSe : δ-FeSe : Fe phase ratio = 25.4 : 73.7 : 0.9% w/w, corresponding to an overall Fe_1.01_Se composition. This finding suggests that an equimolar Fe/Se starting mixture simultaneously forms the two crystal phases in a 1 : 2.9 molar ratio (adding up to slightly increasing in the nominal composition). Similarly, we quantified the excess of iron in the (3 h) *x* = 1.25 and 1.50 materials (*ca.* 8.4 and 13.4% w/w, respectively), which corresponds to an “experimentally retrieved” Fe_1.08_Se and Fe_1.13_Se compositions, revealing a slight decreasing in the nominal composition. This can be associated to losing of material to the environment and/or to large composition range where these iron selenides can exist (0.72 < *x* < 1.02 for δ-FeSe and 1.04 < *x* < 1.08 for β-FeSe).^32,33^

The “post-annealed” (milled for 3 h) materials exhibited sharper Bragg peaks of β-FeSe, though weak and broad peaks of bcc-Fe were still present (*x* = 1.25 and 1.50), no other phase was detected, suggesting that the thermal treatment induced the phase transformation from hexagonal δ-FeSe to β-FeSe, forcing β-FeSe NPs to merge into larger nanocrystals, with average coherent domain sizes above 20 nm. By the other hand, the annealing of *x* = 1.00 sample produced the same phase transition followed by the appearing and/or growing of the monoclinic Fe_3_Se_4_ phase (space group *C*2/*m*). Calorimetry and two series of *in situ* XRPD confirm the presence of Fe_3_Se_4_ phase only for aged samples (see ESI Fig. SI-2(c)†). As transformation of δ-FeSe to Fe_3_Se_4_ implies some release of iron, we interpret the higher stability of the *x* = 1.25 and 1.50 samples by the presence of the extra amount of iron, inhibiting the reaction. Whether this is due to a mass (equilibrium) effect – which for solid-state reactions is hard to envisage – or by the sacrificial behavior of Fe (possibly oxidized by residual O_2_ at high-temperature), cannot be proven, though we favor the second option.

As far as stability issues are concerned, we can point out the appearance of about 17% of Fe_3_Se_4_ formed a few weeks after the ball-milling procedure (*x* = 1.00) and that the diffractograms collected on the 3 h milled materials (for all *x* = 1.00, 1.25 and 1.50 preparations) showed a huge increasing of β-FeSe and proportional decreasing of δ-FeSe Bragg peaks up to few months storage in air, at RT and in the dark. Systematic studies on this ageing effect are in progress and will be presented elsewhere.

Finally, we note that the refined RT cell volumes for the β-FeSe nanophases (77.9 Å^3^, *vs.* 78.7 Å^3^, mean values for the freshly ground and the annealed materials, respectively) accounts for a linear contraction of 1.0% for the small-sized specimen. This in agreement with the common observation of small, but measurable, cell shrinking in metallic nanoparticles at very low crystal sizes,^34^ likely due to the presence of an unsaturated coordination environment, forcing surface atoms to approach the underlying shell(s).

### Comparative structure analysis

Rietveld analysis allowed exploring information about the structure and microstructure of nanocrystalline Fe_*x*_Se samples and this characterization is very important to understand the lack of superconducting properties. The lattice parameters obtained for β-FeSe phase found in all samples are shown in Table 1 that also bring other structural parameters obtained considering 2 different models (with and without refine the occupations and the *z* coordinate of Se). The average values for the lattice parameters of the thermal treated samples are identical to the average values reported for superconducting samples made *via* solid-state and solution methods.^22,35–39^ There is no logical variation of the occupancies and Se coordinate neither with composition nor thermal treatment of the samples, refining to values between 100–105% for Se and from 93% up to 101% to Fe occupation while the Se coordinate varying from 0.256 to 0.271. The as-milled samples presented considerable contraction of the lattice parameters (0.4% in *a* and 0.3 in *c*) in the model that refine occupation and Se coordinate as compared to the values reported in the literature. In the model that occupations and coordinate were fixed a contraction of the lattice parameter *a* (0.4%) and relaxation of 0.3% in *c* were observed. This suggests that β-phase made by mechanical alloying is under compressive stress due to its small crystalline size domain and/or due to coexistence with other crystalline phases. Considerable contraction of the *a* lattice parameter was found for β-FeSe made by solution^35^ and it was attributed for relaxation of surrounding iron atoms in the basal plane into the vacancy. Hsu *et al.*^2^ reported Se deficiency is essential for obtaining superconducting FeSe while Williams *et al.*^3^ have presented straightforward evidence that the correct stoichiometry of Fe_1.01_Se is more important than substantial quantities of selenium vacancies for superconducting tetragonal iron selenide. No evidences of vacant selenium or iron can be sustained by Rietveld analysis results for all stoichiometries tested in present work.

**Table 1:** Structural parameters obtained from Rietveld analysis for as-milled and thermal treated Fe_*x*_Se samples

Sample/ref.	% β-FeSe	SC	*R*_wp_	GOF	*R*-Bragg	*a* (Å)	*c* (Å)	*V* (Å^3^)	*z* (Se)	Occ Fe	Occ Se
36	87	Y	3.99	2.1		3.7685(1)	5.5194(1)	78.38	0.2877	1.01(2)	0.99(3)
40	93.6	N	1.01	1.19		3.7709(1)	5.5216(1)	78.52	0.2669(2)	1	1
40	100	N	1.21	1.11		3.7711(1)	5.5214(1)	78.52	0.2672(2)	1	1
41	—	?				3.775	5.527	78.76	0	1	1
42	—	?				3.773	5.529	78.71	0.26	1	0.88
35	100	Y	6.12	0.94		3.7735(1)	5.5223(1)	78.635(2)	0.2653(1)		
35	100	Y	5.48	1.00		3.7723(1)	5.5225(1)	78.588(2)	0.2656(1)		
43	—	?				3.6007	5.8715				
37	—	N	7.17			3.7720(9)	5.5161(5)	78.480(11)	0.2670		0.975
38	90.7	Y				3.7622(2)	5.5018(5)		0.2624(1)		
22	—	Y				3.7796(3)	5.5193(5)				
39	—	Y				3.76966	5.5201				
44	100	Y	9.28	2.50	3.78	3.8280(2)	5.5821(3)		0.2676(1)	1	1

**Model 1**											
Fe_1.00_Se			0.89	2.15	0.509	3.7657	5.5052	78.067	0.26	1	1
Fe_1.25_Se			0.69	1.60	0.197	3.7545	5.5146	77.735	0.26	1	1
Fe_1.50_Se			0.64	1.51	0.172	3.7507	5.5135	77.561	0.26	1	1
tt-Fe_1.00_Se			3.55	3.41	1.635	3.7748	5.5189	78.640	0.26	1	1
tt-Fe_1.25_Se			1.85	2.08	2.042	3.7734	5.5208	78.608	0.26	1	1
tt-Fe_1.50_Se			1.48	1.73	1.347	3.7743	5.5205	78.643	0.26	1	1

**Model 2**											
Fe_1.00_Se			0.75	1.83	0.380	3.7666	5.5029	78.075	0.2687	0.93	1.01
Fe_1.25_Se			0.69	1.60	0.196	3.7544	5.5146	77.734	0.2599	1.01	0.99
Fe_1.50_Se			0.60	1.42	0.149	3.7578	5.5173	77.913	0.2564	0.97	1.01
tt-Fe_1.00_Se			3.34	3.22	0.397	3.7755	5.5190	78.672	0.2655	0.80	1.05
tt-Fe_1.25_Se			1.34	1.52	0.594	3.7746	5.5220	78.675	0.2710	0.93	1.02
tt-Fe_1.50_Se			1.31	1.54	0.541	3.7749	5.5212	78.677	0.2685	0.99	1.00

### Size and strain analysis

Fig. 1(c) shows the average crystallite size and the microstrain of the phases as a function of the composition *x* of the samples. The average crystallite size of the phases observed for the as-milled samples is around 15 nm and after annealing these values grows up 22 nm for β-FeSe phase and up to 29 nm for residual iron. The microstrain changes a little with composition and the mean values obtained were about 1.7% for the δ-FeSe and 1.8% for the β-FeSe phase, which reveal high degree of disorder in the as-milled samples. The microstrain decreases to about 1.1% for the β-FeSe phase after thermal treatment, in agreement with the expectation to have less defective structure as crystallite size increases. However, the microstrain increases for minority phases (δ-FeSe and residual Fe), since them are more susceptible to tensile and/or compressive stress.

### Microscopic characterization

**Fig. 2:**
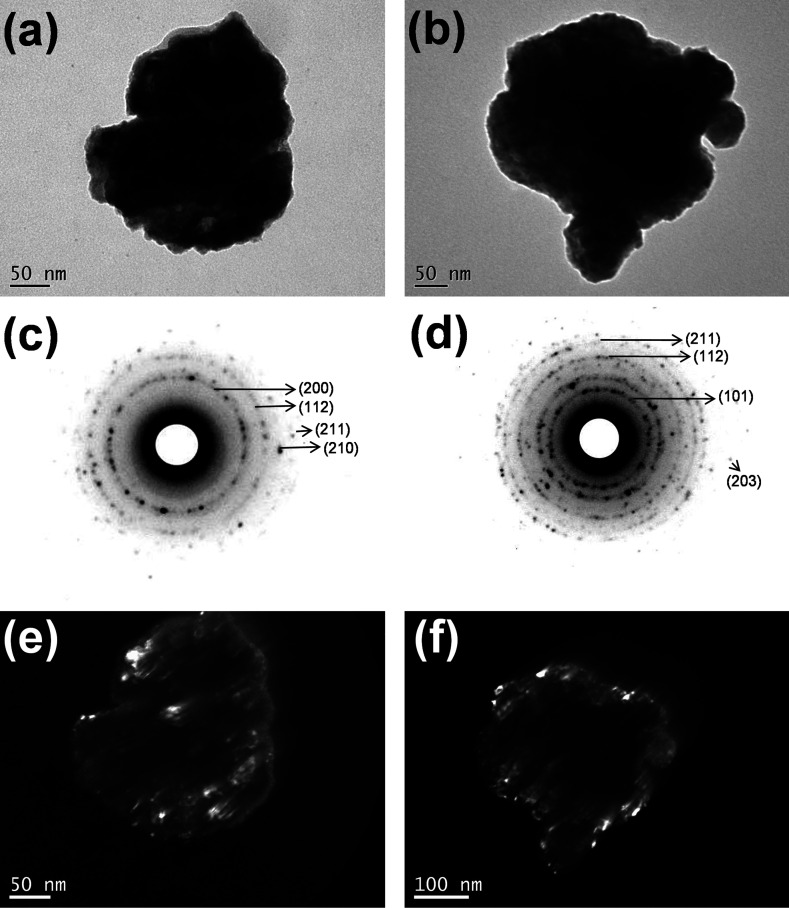
TEM images of (a) as-milled and (b) post-annealed Fe_1.25_Se sample. The SAED patterns (c) and (d) and dark field (e) and (f) for the samples. The Miller index in panels (c) and (d) are from the β-FeSe phase.

Once the Fe_1.25_Se sample gave the highest β-FeSe phase fractions (after thermal treatment), several TEM images on different portions of the sample were collected, showing that the Fe_1.25_Se samples (as-milled and thermal treated) are formed by complex aggregates of irregular shape, about 350 to 400 nm in size. Representative images are shown in Fig. 2(a) and (b). Within these aggregates, the presence of much smaller (and randomly oriented) crystallites, with sizes in the 10–30 nm range, is imaged, in agreement with XRPD data analysis. The agglomeration of the particles/crystallites can be understood by the cold-welding mechanism (that compete with fracture mechanism) inherent to the mechanical alloying process. The SAED patterns shown in Fig. 2(c) and (d) can be indexed by the β-FeSe phase in agreement to that observed by XRPD and attest that the samples are really nanocrystalline even after thermal treatments at 400 °C. The random orientation and size of the crystallites are further supported by the Debye rings visible in SAED patterns and by the dark field images (Fig. 2(e) and (f)).

At variance, a few regularly shaped and faceted particles with about 100 × 80 nm in size were found in the TEM images of the Fe_1.25_Se sample milled for 3 h and one representative image is shown in the ESI (Fig. SI-3(a)†). The SAED pattern shown in Fig. SI-3(b)† indicates that these particles are single-domain nanocrystals. Their SAED patterns and corresponding dark field images (Fig. SI-3(c)†) allow one to see the crystallographic texture of the nanometric single crystal-like particles. This result calls attention for the large size distribution of the mechanical alloyed samples that can also be affecting its superconducting behavior.

### Magnetic and electrical characterization

The magnetization of the as-milled and thermal treated Fe_*x*_Se (*x* = 1.00, 1.25 and 1.50) samples up to magnetic fields of 20 kOe at room temperature are shown in the ESI (Fig. SI-4†). The as-milled Fe_1.00_Se sample has a ferromagnetic behavior, with saturation of 34 emu g^−1^ at 10 kOe, 100 Oe coercivity and 2.42 emu g^−1^ of remanent magnetization, which can be attributed to the presence of the δ-FeSe phase and/or residual Fe. After thermal treatment this sample showed typical ferrimagnetic behavior characterized by absence of saturation up to field of 20 kOe, with maximum magnetization of 4 emu g^−1^, coercivity of the 575 Oe and remanent magnetization of de 0.99 emu g^−1^. According to XRPD data the thermal treatment promotes a phase transition from hexagonal δ-FeSe to monoclinic Fe_3_Se_4_ and vanishing of residual iron, then the magnetic response of the thermal treated Fe_1.00_Se sample can be strongly influenced by the Fe_3_Se_4_ phase that is reported as ferrimagnetic and in the nanometric form presents unusually high values of coercivity (25 kOe) attributed to uniaxial magnetocrystalline anisotropy with ordered cation vacancies.^45^

The hysteresis loops of the Fe_1.25_Se and Fe_1.50_Se samples before and after thermal treatment are very similar in shape and can be associated to ferromagnetic character of theses samples, which presents saturation values of 13 emu g^−1^ and 30 emu g^−1^, remanent magnetization of 4 emu g^−1^ and 8.5 emu g^−1^, respectively, and almost identical coercivity of ∼600 Oe. The difference in the saturation and remanent magnetizations can be attributed to the differences of residual iron content in each sample, 8.4% and 13.4% for Fe_1.25_Se and Fe_1.50_Se samples, respectively. The magnetic behavior of the thermal treated samples cannot be associated to δ-FeSe once it was not observed in the XRPD (Fig. 1(a)), suggesting that the β-FeSe phase and residual iron govern the magnetic properties of these samples.

**Fig. 3:**
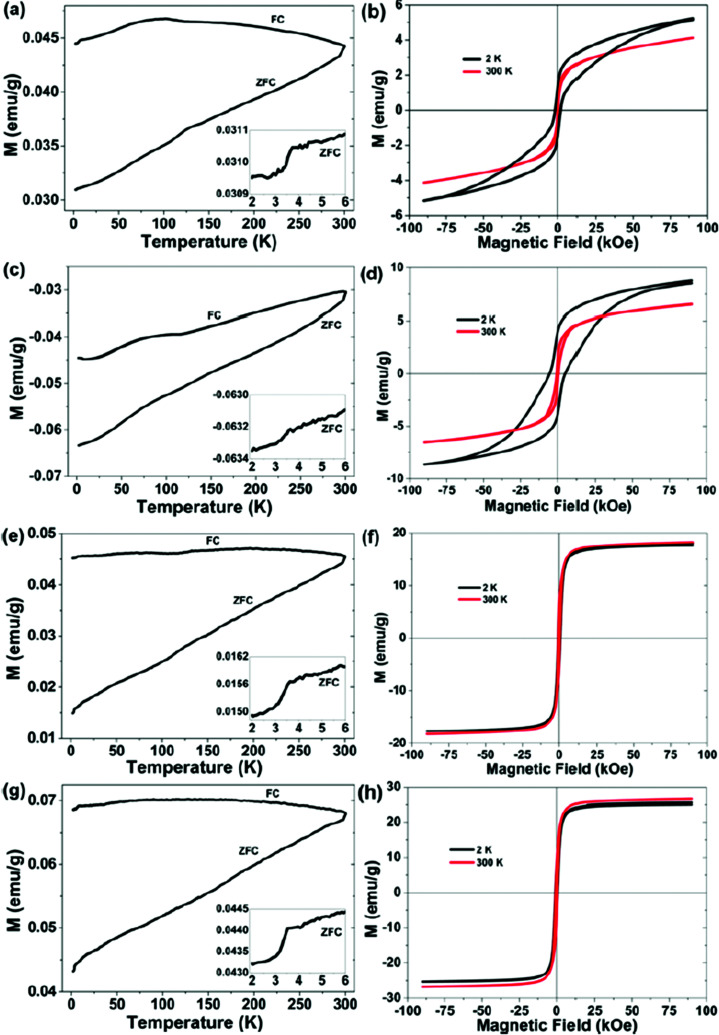
Temperature dependence of the magnetization (a) and hysteresis loops (b) for the as-milled Fe_1.00_Se sample, thermal treated samples: Fe_1.00_Se ((c) and (d)), Fe_1.25_Se ((e) and (f)), and Fe_1.50_Se ((g) and (h)).

Magnetic hysteresis loops of the Fe_*x*_Se (*x* = 1.00, 1.25 and 1.50) samples were measured at different temperatures in an applied field as higher as 90 kOe (Fig. 3). These experiments were performed two months after samples production. The hysteresis loops for the Fe_1.25_Se and Fe_1.50_Se samples are in agreement with those collected for smaller magnetic fields (Fig. SI-4†) and showed little influence of the temperature. On the other hand, hysteresis loop for the as-milled Fe_1.00_Se sample showed a huge difference as compared to the results collect up to 20 kOe just after of the sample production; while for the thermal treated samples similar results were observed. The reduction of the magnetic saturation to 4 emu g^−1^ and the increasing of coercivity to 500 Oe for the aged as-milled Fe_1.00_Se sample can be attributed to the appearing of the ferrimagnetic Fe_3_Se_4_ phase and reduction of hexagonal δ-FeSe content. These phase transitions from δ-FeSe to β-FeSe and Fe_3_Se_4_ were also induced by thermal treatment of this sample as shown in XRPD pattern (v) in Fig. 1, explaining why the magnetic measurements for the thermal treated sample are reproducible. The magnetic loops for Fe_1.00_Se sample showed strong dependence with temperature revealing broad hysteresis that do not reach saturation even for 90 kOe field at 2 K, which is comparable to the hysteresis loops reported for monoclinic Fe_3_Se_4_ phase (ref. 45) and β-FeSe nanocacti.^46^ Modestly high coercivity was observed for the Fe_1.00_Se sample, which increases with temperature decreasing from 0.2 kOe (300 K) to 1.9 kOe (2 K) for the as-milled sample and from 0.5 kOe (300 K) to 5.0 kOe (2 K) for the thermal treated one (Fig. 3(a)). The lack of high coercivity reported for nanocrystalline Fe_3_Se_4_ phase (ref. 45) can be understood by the fact that it coexist with tetragonal β-FeSe in the Fe_1.00_Se samples.

The temperature dependence of magnetization of as-milled and thermal treated Fe_1.00_Se, Fe_1.25_Se and Fe_1.50_Se samples were carried out by zero-field-cooled (ZFC) and field-cooled (FC) measurements, also shown in Fig. 3. The magnetization values are positive for all samples except for thermal treated Fe_1.00_Se (that according to XRPD data presents only β-FeSe and Fe_3_Se_4_ phases). The positive values of magnetization can be attributed to the existence of residual Fe in the samples, which contributes to the ferromagnetic signal. Significant splitting between ZFC and FC measurements are observed for all samples (except for thermal treated Fe_1.00_Se), confirming that magnetic behavior of these samples are governed by the β-FeSe and ferromagnetic impurities, mainly residual iron and/or δ-FeSe phase and/or other ferromagnetic phase with quantity too small to detect by other methods of characterization.^35,38^ A slight magnetic anomaly was observed in the FC curves of all samples, being more intense in Fe_1.00_Se samples, where a broad peak can be observed around 100 K. Moreover, these FC's also shows abrupt reduction of magnetization at about 7 K, while ZFC measurements showed similar feature at about 3.5 K for all samples, which can be associated to poor superconducting transition.

A similar loss of superconductivity and appearance of antiferromagnetism was observed in the β-FeSe produced from solution and solid-state methods.^35,46^ The exposure to oxygen or water present in the atmosphere or solvent will suppress superconductivity and induce antiferromagnetic behavior, even if the exposure is postsynthetic. It is likely that the oxygen atoms induce a strong localized magnetic moment on the neighboring Fe atoms, and these moments prevent the onset of superconductivity. This may explain why other samples of β-FeSe produced from solution have not exhibited superconductivity.^35^ The absence of the superconductivity in β-Fe_*x*_Se nanosheets and nanocactis was attributed to small size of the nanostructures with a thickness far smaller than its penetration depth.^46^

Electrical resistance measurements of as-milled and thermal treated Fe_1.25_Se samples (Fig. SI-5†) showed an unexpected insulator-like behavior where the resistance increases with temperature decreasing. This effect was observed for FeSe samples produced by solution methods and attributed to oxygen or water incorporated directly into the structure, where the oxygen atoms induce a strong localized magnetic moment on the neighboring Fe atoms, and these moments prevent the onset of superconductivity.^35^ The resistance values for the thermal treated sample is one order of magnitude smaller than before annealing, fact that can be correlated to the hexagonal δ-FeSe phase disappearance, as shown by XRPD data, and/or to the decreasing of defects and the growing of crystalline size domains of β-FeSe phase. The step-like increasing of the resistance with temperature decreasing can be associated to semiconducting behavior, while a visible drop of resistance at 4 K confirms the poor superconducting behavior of this sample.

## Conclusions

We showed that considerable quantity of tetragonal β-FeSe phase can be obtained by mechanical alloying using high-energy ball mill for only 3 hours (no further processing need). The compositions tested (Fe_1.00_Se, Fe_1.25_Se and Fe_1.50_Se) resulted in 29%, 19% and 12% of the β-FeSe phase, which presented significant quantity increasing (∼93%) after 1 hour of heat treatment at 400 °C under vacuum. Effects due to iron and selenium vacancies, lattice contraction were considered, but none straight correlations with neither composition nor thermal treatment were observed. A broad size distribution of the β-FeSe nanocrystalline domains was evidenced by TEM experiments. Poor superconductivity was observed and it can be due to ferro/ferrimagnetic impurity phases, such as residual Fe, hexagonal δ-FeSe phase and monoclinic Fe_3_Se_4_, but we conclude that the high microstrain jointly the small average size and large size distribution of the nanocrystalline domains of the tetragonal β-FeSe phase are the main reasons for the lack of superconductivity in the mechanical alloyed samples. The electrical and magnetic properties of nanoparticle samples are not only intrinsic to the particles but also depend on the conditions in which the particles are arranged and its own microstructure. As it is well know some of the highest critical temperatures in Fe-based superconductors have been achieved by *in situ* or postsynthetic intercalation of molecular spacer layers into β-FeSe. The mechanochemical process can be assisted by some process control agents (wet mechanical alloying) that allow for *in situ* intercalation of spacer layers, which may lead to the discovery of new compounds with improved physical properties. Moreover, mechanical alloying utilizes nontoxic and inexpensive starting materials, occurs at a room temperature, and is easily scalable; it may be mainly attractive for future technological applications.

## Conflicts of interest

There are no conflicts to declare.

## Supplementary Material

RA-008-C7RA13473H-s001
