# Supplementary material for: Nanosized tetragonal β-FeSe phase obtained by mechanical alloying: structural, microstructural, magnetic and electrical characterization
Source: RSC Adv. 2018 Feb 20;8(15):8190–8. doi: 10.1039/c7ra13473h (PMC9078550; doi:10.1039/c7ra13473h)
Supplement: RA-008-C7RA13473H-s001 [file RA-008-C7RA13473H-s001.pdf]

Electronic Supplementary Material (ESI) for RSC Advances.
This journal is © The Royal Society of Chemistry 2018

RSC Advances

Electronic supplementary information

Nanosized Tetragonal β-FeSe phase obtained by Mechanical Alloying: Structural, Microstructural, Magnetic and Electrical Characterizations

K.F. Ulbrich[a] and C.E.M. Campos[a]

[a.] *Departamento de Física, Universidade Federal de Santa Catarina, Campus Trindade, 88 040-900 Florianópolis, Santa Catarina - SC, Brazil.*

E-mail: carlos.campos@ufsc.br

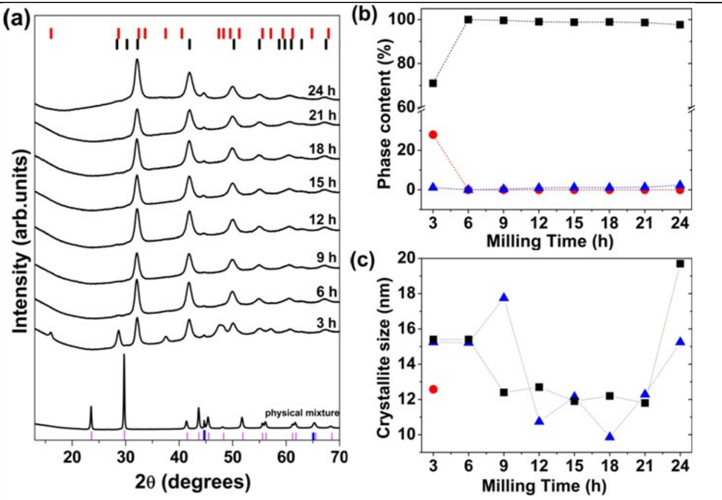

Figure SI-1. (a) XRD patterns of the $Fe_{1.0}Se$ sample as a function of milling time. (b) Phase fractions and (c) Average crystallite size for δ-FeSe, β-FeSe and residual Fe phases versus milling time. The black symbols are for representing the δ-FeSe phase, the red ones for the β-FeSe phase and blues are for residual Fe.

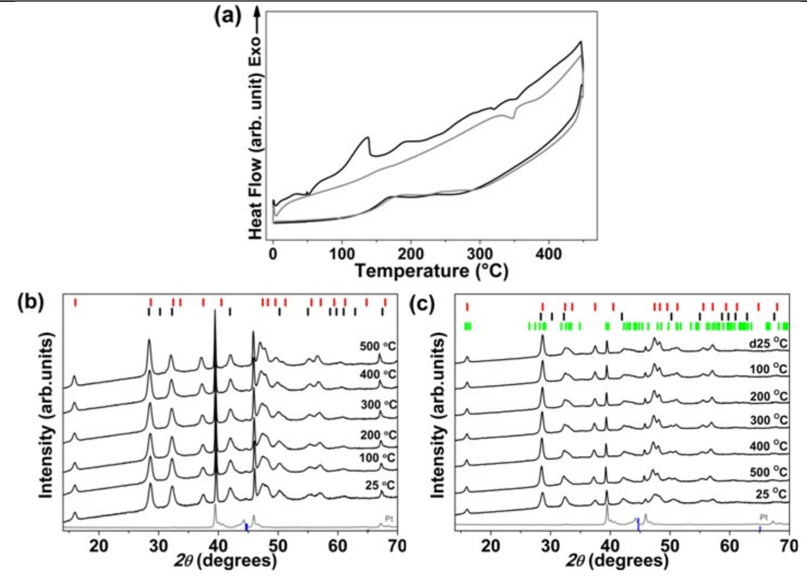

Figure SI-2. (a). First and second runs (heating and cooling) DSC curves of $Fe_{1.0}Se$ sample milled. The gray lines represent the second run. XRPD patterns of the of the as-milled $Fe_{1.0}Se$ sample as a function of temperature collected 21 days (b) and 4 months (c) after its production.

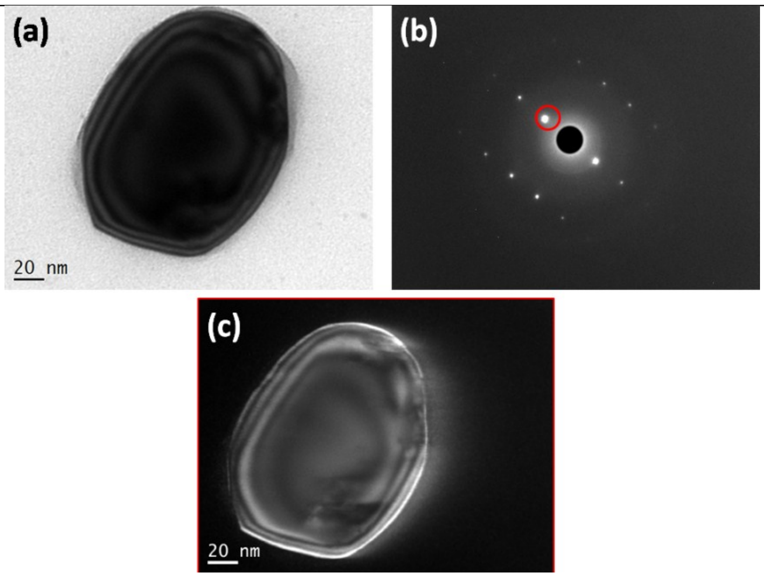

Figure SI-3. TEM image (a) of as-milled $Fe_{1.25}Se$ sample and its SAED pattern (b) and dark field image (c).

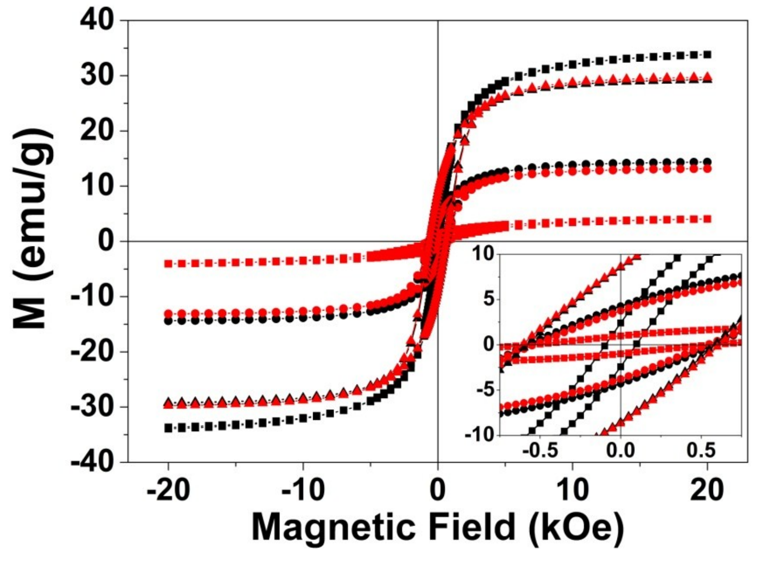

Figure SI-4. Magnetizations curves for $Fe_{1.0}Se$ (squares), $Fe_{1.25}Se$ (circles) and $Fe_{1.5}Se$ (triangles) samples at room temperature. Black and red symbols represent the values for the as-milled and thermal treated

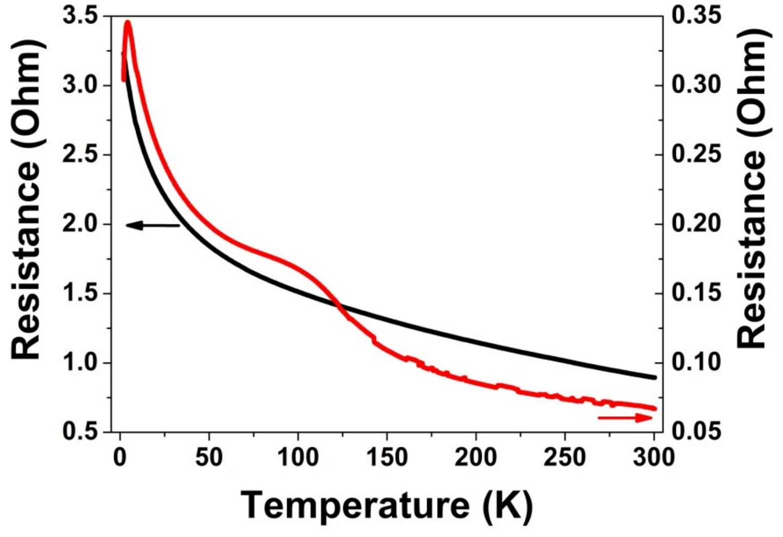

Figure SI-5. Electrical resistance of as-milled (black line) and thermal treated (red line) $Fe_{1.25}Se$ samples as a function of temperature.
